# Strategies for MCR image analysis of large hyperspectral data-sets

**DOI:** 10.1002/sia.5040

**Published:** 2012-05-22

**Authors:** David J Scurr, Andrew L Hook, Jonathan A Burley, Philip M Williams, Daniel G Anderson, Robert C Langer, Martyn C Davies, Morgan R Alexander

**Affiliations:** a Laboratory of Biophysics and Surface Analysis, University of NottinghamNottingham, NG7 2RD, UK; b David H. Koch Institute for Integrative Cancer Research, Massachusetts Institute of Technology77 Massachusetts Avenue, Cambridge, MA, 02139, USA

**Keywords:** time-of-flight secondary ion mass spectrometry, multivariate curve resolution, microarray, high-performance computing

## Abstract

Polymer microarrays are a key enabling technology for high throughput materials discovery. In this study, multivariate image analysis, specifically multivariate curve resolution (MCR), is applied to the hyperspectral time of flight secondary ion mass spectroscopy (ToF-SIMS) data from eight individual microarray spots. Rather than analysing the data individually, the data-sets are collated and analysed as a single large data-set. Desktop computing is not a practical method for undertaking MCR analysis of such large data-sets due to the constraints of memory and computational overhead. Here, a distributed memory High-Performance Computing facility (HPC) is used. Similar to what is achieved using MCR analysis of individual samples, the results from this consolidated data-set allow clear identification of the substrate material; furthermore, specific chemistries common to different spots are also identified. The application of the HPC facility to the MCR analysis of ToF-SIMS hyperspectral data-sets demonstrates a potential methodology for the analysis of macro-scale data without compromising spatial resolution (data ‘binning’). Copyright © 2012 John Wiley & Sons, Ltd.

## Introduction

Many applications of materials in biomedicine suffer from suboptimal performance, such as the high incidence of catheter-associated urinary tract infections. In these cases, new materials are required that have properties ideally suited to the application; in the case of urinary catheters, the material must be both antibacterial and flexible. Polymer microarrays are ideally suited to high throughput materials screening by presenting thousands of unique polymers on one glass microscope slide.^1^ Combinatorial microarrays have been used to screen for biomaterials that are capable of supporting the clonal expansion of stem cells, resist bacterial attachment, identify switchable materials and sort co-culture cell populations.^2^–^6^ Furthermore, high throughput surface characterisation of arrays has successfully been applied to determine the chemical and physical properties of the materials^7^–^10^ which can then be correlated with the biological performance of the materials to elucidate structure–function relationships.^2^,^3^ Progress in this field relies on the application of polymer microarrays, with an expansion of the combinatorial space that these explore, and increased throughput in processing tools to effectively analyse the plethora of data that high throughput studies produce.

Time of flight secondary ion mass spectroscopy (ToF-SIMS) is a surface characterisation technique with the capacity to readily analyse materials ranging across electronics, metallic, polymer and biological samples.^11^–^13^ The volume of data associated with ToF-SIMS hyperspectral image analysis can sometimes lead to difficulty in data handling and interpretation. This is particularly notable when performing comparative studies upon multiple samples, such as microarray systems. MVA techniques have proven vital in extracting the important aspects from data acquired from such systems.^12^ Moreover, the MVA technique, multivariate curve resolution (MCR), has successfully analysed complex hyperspectral image data-sets from carbohydrate and polymer/drug microarrays.^14^,^15^ These studies have demonstrated a capacity to discern specific features within individual array printed spots as well as the chemical heterogeneities from different printed spots located across larger array areas.

Although techniques such as MCR can alleviate some of the ‘manual’ workload associated with ToF-SIMS data analysis, certain systems such as microarrays can still pose a challenge because of the number of separate samples (spots) involved and/or because it is desirable to analyse mm-scale areas. Both of these approaches result in large data-sets. The current computing power of commonly employed desktop computers often requires data to be reduced (binned) for MCR image analysis. This limits the potential to analyse multiple samples or mm-scale regions at high resolution, which can be routinely achieved using the stage scan ‘image stitching’ functionality of SurfaceLab 6 (IONTOF GmbH). The production of spots is not flawless as the printing can sometimes form spots which are not homogeneous mixtures of the monomer constituents which must be detected and analysed by ToF-SIMS. This study aims to demonstrate a method for automated cross-comparison of individual data-sets by analysing multiple data-sets as a single entity.

## Experimental

### Array printing

Arrays were prepared as previously described.^16^ Prior to printing, epoxy-coated glass slides (Genetix) were prepared by dip-coating with a 4% (w.v) poly(2-hydroxyethyl methacrylate) (pHEMA) solution in ethanol. The polymer microarrays were produced onto the pHEMA-coated glass slides using a contact printer (Biodot). The environment throughout printing was maintained at O_2_ < 1300 ppm, 25 °C and 40% relative humidity. Slotted metal pins (946MP6B, Arrayit) with a diameter of 220 µm were used to transfer approximately 2.4 nL of monomer solution (75 % (v/v) monomer in DMF with 1 % (w/v) photoinitiator 2,2-dimethoxy-2-phenylacetophenone) before irradiating with a long wave UV source for 10 s. Once produced, the resulting arrays were dried at < 50 mTorr at 25 °C for seven days.

### ToF-SIMS

Measurements were conducted using a ToF-SIMS IV (IONTOF GmbH) instrument using a 25 kV Bi_3_^+^ primary ion source operated with a pulsed target current of ∼ 1 pA. The primary ion beam was rastered over analysis areas of 500 × 500 µm, capturing data from whole individual array spots and some surrounding pHEMA background at a resolution of 256 × 256 pixels. An ion dose of 2.45 × 10^11^ ions/cm^2^ was applied to each sample area ensuring static conditions were maintained throughout. Both positive and negative secondary ion spectra were collected (mass resolution of >10,000), over an acquisition period of 15 scans (the data from which were added together). Owing to the non-conductive nature of the samples, charge compensation, in the form of a low energy (20 eV) electron floodgun, was applied.

### Multivariate data analysis

Two distinct peak lists, comprising 461 and 417 peaks, were generated for the positive and negative ToF-SIMS data, respectively. These lists were created based on eight separate sample data-sets and were used to retrospectively reconstruct the image data. In this study, only the positive data will be discussed. This data was subsequently exported and processed simultaneously using PCA^17^,^18^ (R package version 1.24.0) and MCR^18^,^19^ (R package version 0.0.4, modified as below). The PCA analysis was used as a pre-curser to the MCR analysis, and an evaluation of the ‘scree’ plot (SI. 1) was used to help establish the number of MCR components to apply. In order to validate the appropriate number of components to apply for MCR analysis, MCR analysis was performed and the results assessed for a range of component numbers. In both the PCA and MCR analysis, no data pre-treatments were applied. The Alternating Least-Squares MCR (ALS-MCR) was undertaken using random initial estimates of the scores and loadings. Since the deconvolution results are partially dependent upon the initial starting estimates, each ALS-MCR analysis was repeated ten times from different starting points. The resulting ten sets of loadings data of each component were then *k*-means clustered and the mean of each cluster used as the initial guess for one final round of ALS-MCR, again using random scores.

Owing to the large size of the data-set and the multiple repeats required, the ALS-MCR analysis was undertaken on the distributed memory High-Performance Computing (HPC) cluster at the University of Nottingham. The MCR R package was modified to increase performance and exploit the multiple cores of each of the compute nodes, with an order-of-magnitude decrease in the time to one solution achieved. As the ten ALS-MCR analyses for each number of components were performed concurrently using the HPC cluster, the total wall-time for the analysis was less than one one-hundredth of what it would have been using our desktop machine.

## Results and discussion

**Figure 1:**
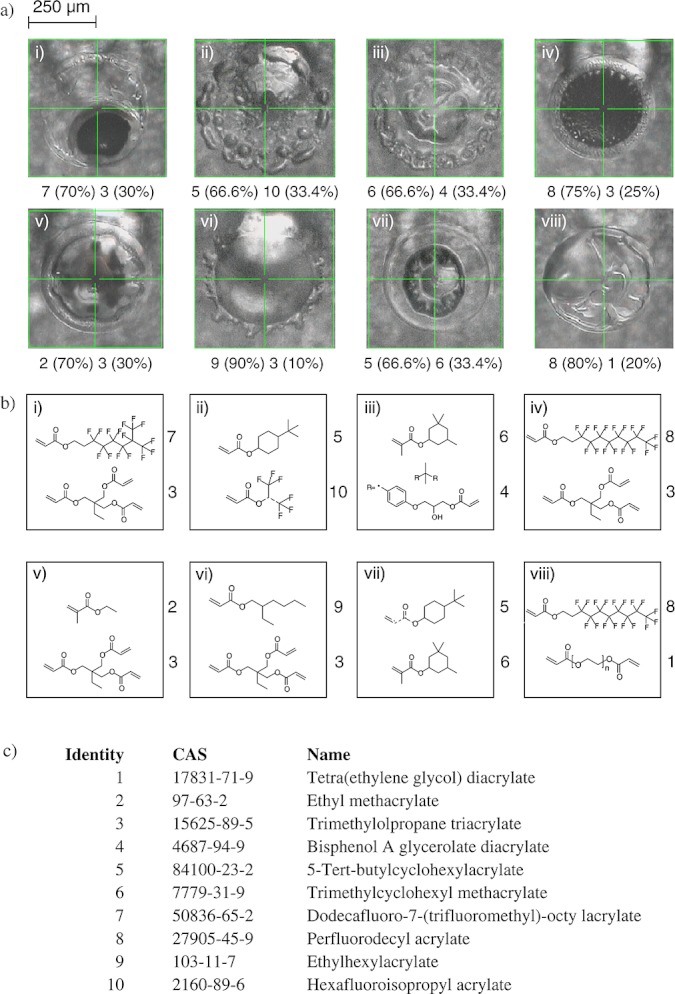
a) Optical images of the eight individual polymer spots investigated in this study and their monomer composition, b) specific monomer structures and c) table of monomers, where the numbers listed within 1a and b correspond to listed monomer identities.

The hyperspectral images from eight polymer spots, chosen from a 576 spot array because they exhibited chemical heterogeneities, were analysed by MCR. In many cases, the spot appearance observed by optical microscopy was non-uniform as shown in Fig. 1a. The constituent monomers of these spots are shown in Figs. 1b and c. High spatial resolution ToF-SIMS image data, 256 × 256 pixels, over an area of 500 × 500 µm were acquired from each spot which was subsequently collated for analysis as a single data-set. The ‘scree’ plot of the PCA analysis of this data-set (SI. 1) does not identify a definitive number of components to apply, but suggests a value ranging from 7 to 12. MCR image analysis was then performed using different numbers of components with the results examined for evidence of ‘over’ fitting, where similar spatially located features are identified with similar associated secondary ions for multiple components. Using this methodology, a component number of 9 was established as being the most appropriate to analyse the data.

The scores image, corresponding loadings plot and an associated table of the most significantly loaded ions are shown in full for each of the nine components in the supplementary information (SI. 2a – i). This nine component MCR image analysis clearly identified the pHEMA coating of the microscope slide, mutual chemistries across different spots that corresponded to common monomer constituents, as well as some sample contamination. The lateral resolution of these images also allows for the observation of the distribution of each component within individual spots.

**Figure 2:**
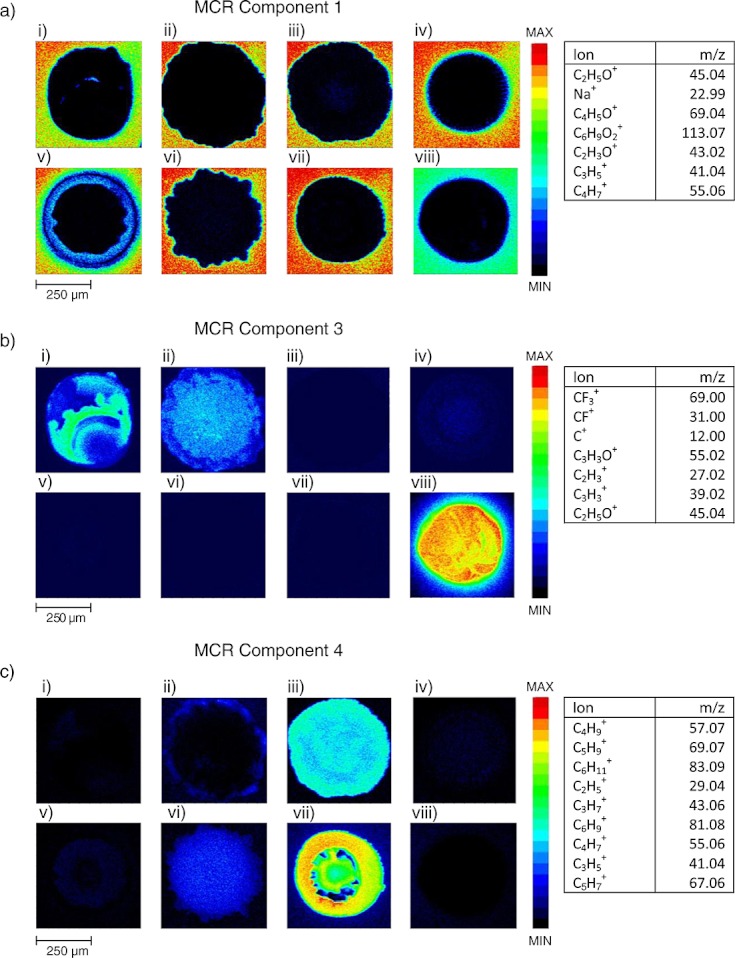
Scores image and significant loadings data for MCR components a) 1, b) 3 and c) 4, where the sample layout corresponds to that illustrated in Fig. 1.

The scores image and most significant loadings for MCR component 1 are shown in Fig. 2a, where it is clear from the lateral distribution within each of the eight images that this component identifies the pHEMA substrate material. This assessment is confirmed through an analysis of the loadings of MCR component 1 where the three highest loaded secondary ions are C_2_H_5_O^+^, Na^+^ and C_4_H_5_O^+^. The C_2_H_5_O^+^ and C_4_H_5_O^+^ secondary ions are characteristic of pHEMA.^20^ The Na^+^ originates either as a contaminant in the pHEMA or the ethanol used in the dip-coating procedure. Although in an individual data-set, the identification of the substrate material is often trivial, establishing such a clear substrate component in this single analysis of multiple spots is a significant validation of the MCR methodology on this consolidated data-set.

The spot regions highlighted in components 3, 4, 7, 8 and 9 (SI. 2c, d, g, h and i) correspond to specific monomer constituents within the spots. The scores image for component 3 highlights spots i, ii, iv and viii (Fig. 2b), where the two highest loaded ions are CF_3_^+^ and CF^+^, both of which are characteristic of the fluorine containing monomers present in these spots. The intensity of spot iv appears significantly lower than anticipated (Fig. 2b) due to the ‘masking’ of the spot chemistry by polydimethylsiloxane (PDMS) contamination. This is confirmed by the scores image for component 2 which covers much of spot iv (SI. 2b), where the most significantly loaded ion, SiC_3_H_9_^+^, is characteristic of PDMS. Individual monomers are co-localised with high intensity regions in components 4, 7 and 8. The scores image for component 4 is shown as an example in Fig. 2c, highlighting spots iii and vii which both contain the trimethylcyclohexyl methacrylate monomer.

Recent technical advances have enabled high resolution ToF-SIMS data to be acquired over areas of many square millimetres, however, conventional computing will be incapable of performing image MVA techniques on the resulting data-sets. Consequently, high-performance computing facilities are required. Assuming the same number of peaks, 461 in this instance, and the same lateral resolution, the size of the data-set analysed in this study is the equivalent of a data-set obtained over a 1 × 2 mm area. This study demonstrates the potential to analyse the large ToF-SIMS hyperspectral data-sets which could be obtained from a full microarray using MCR, either as an individual macro-scale analysis or as a series of consolidated data-sets analysed together as a single entity.

## Conclusions

This study has demonstrated for the first time that the MVA technique of imaging MCR can be transferred to analysing numerous image data-sets as a single entity. Whilst anticipated outcomes such as the differentiation of the substrate material are clear, more specific spot-to-spot chemical heterogeneities have also been observed whilst maintaining each individual analysis region's full resolution. The use of the HPC facility vastly increased the throughput of data analysis and also demonstrates a method for the analysis of macro-scale sample regions with no reduction in the volume of data.
