# Supplementary material for: Strategies for MCR image analysis of large hyperspectral data-sets
Source: Surf Interface Anal. 2012 May 22;45(1):466–70. doi: 10.1002/sia.5040 (PMC3579489; doi:10.1002/sia.5040)
Supplement: Supplementary file 1 [file sia0045-0466-SD1.pdf]

SI. 1) Scree plot of PCA analysis

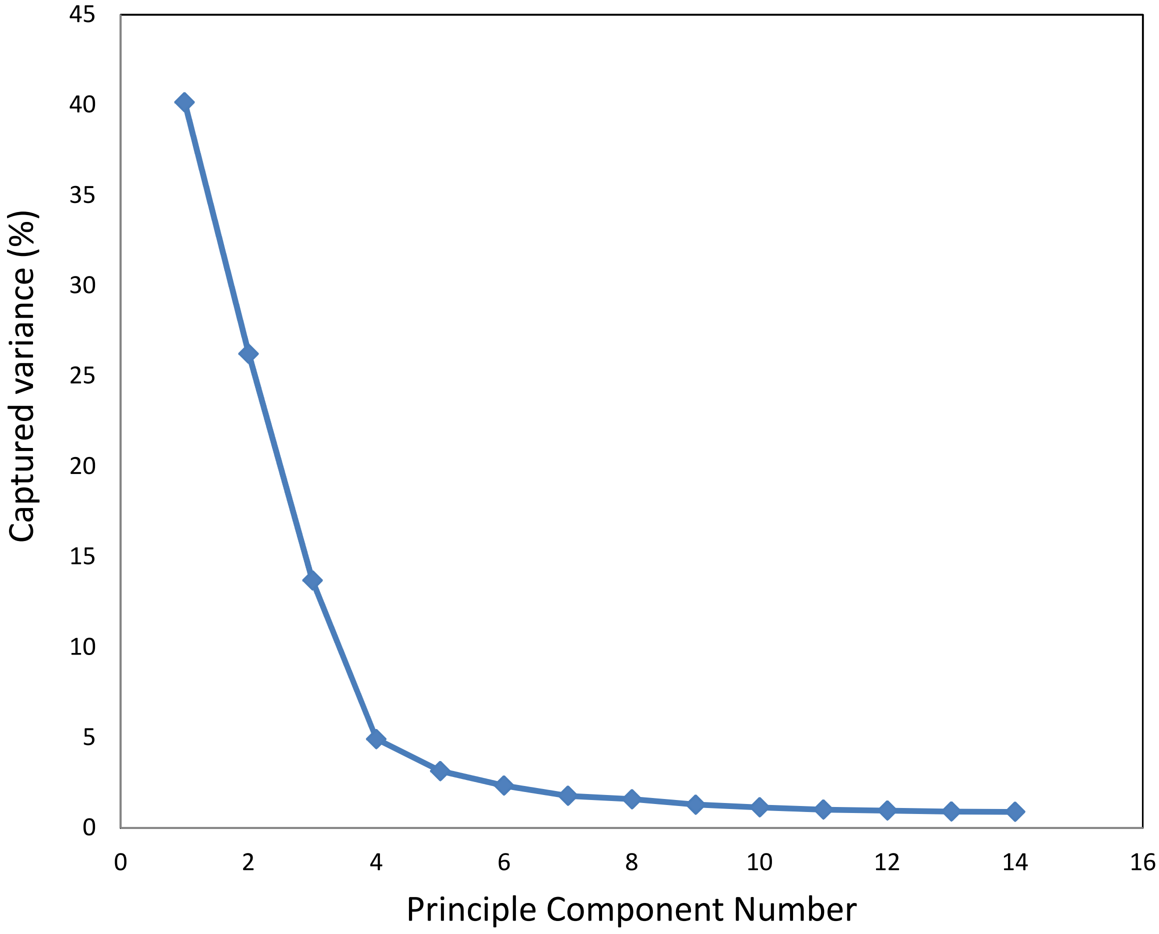

SI. 2a) PC1

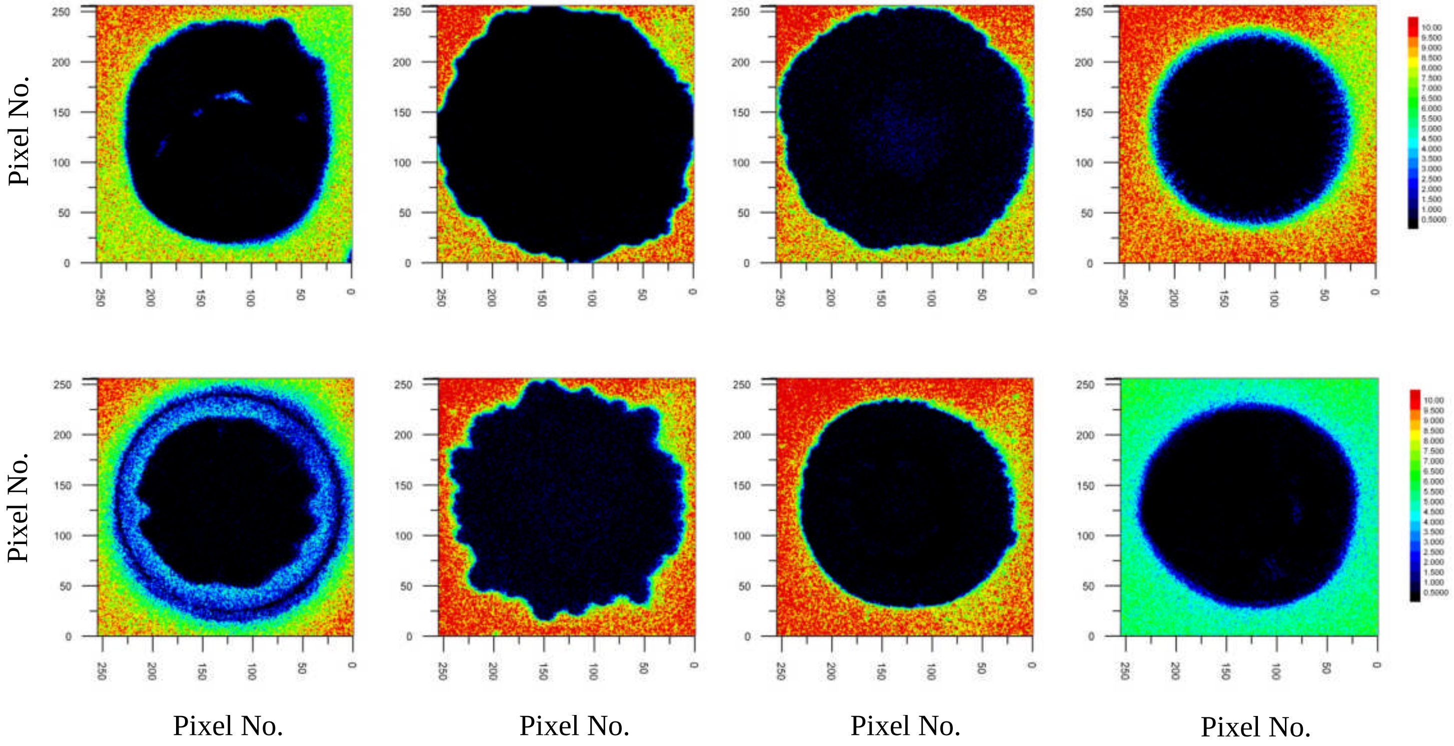

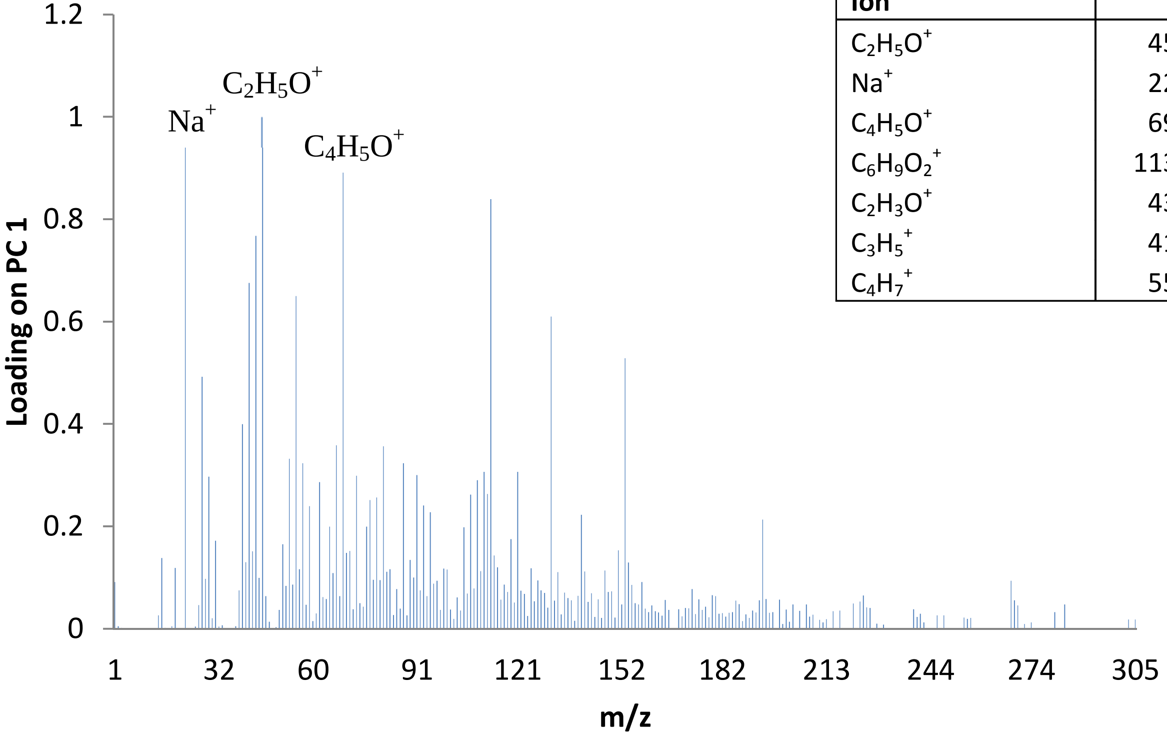

Ion	m/z
$C_2H_5O^+$	45.04
Na^+	22.99
$C_4H_5O^+$	69.04
$C_6H_9O_2^+$	113.07
$C_2H_3O^+$	43.02
$C_3H_5^+$	41.04
$C_4H_7^+$	55.06

SI. 2b) PC 2

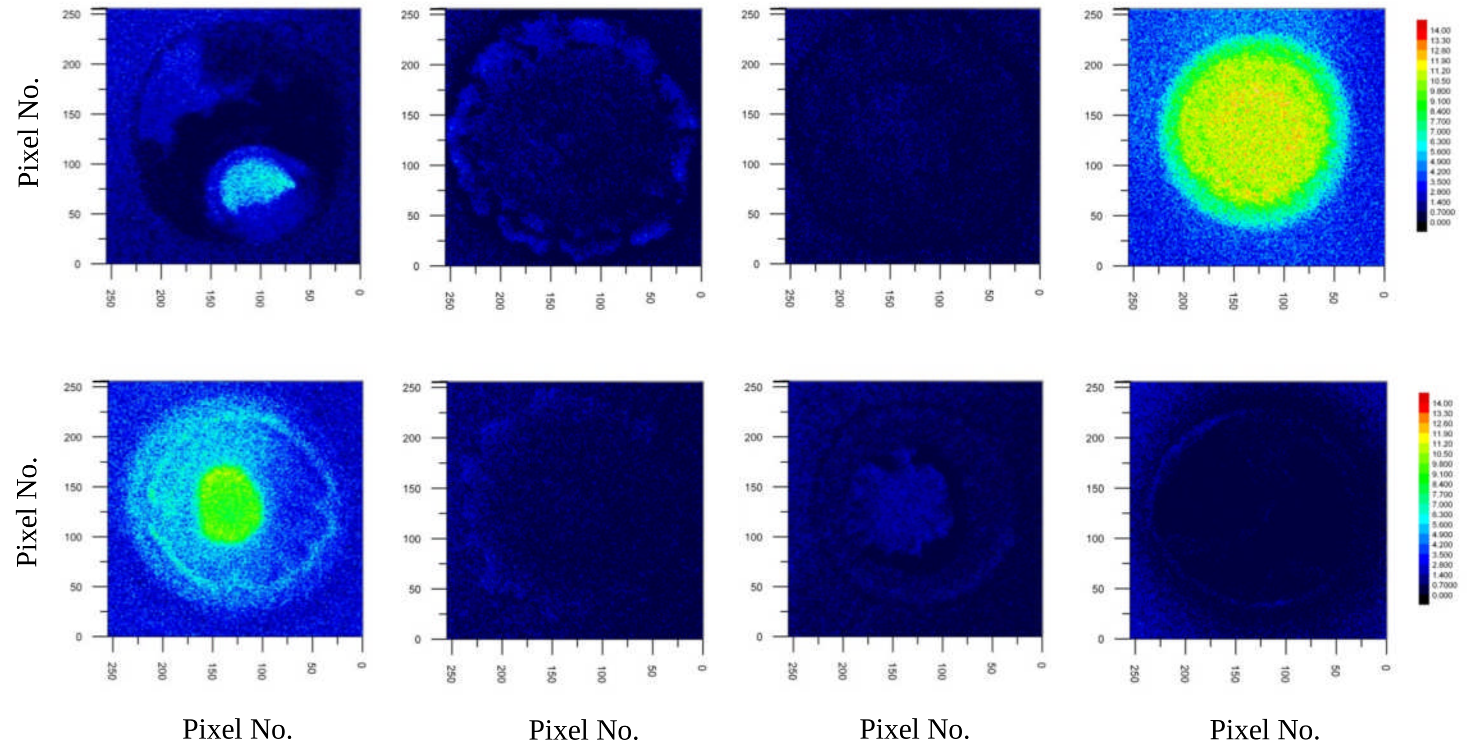

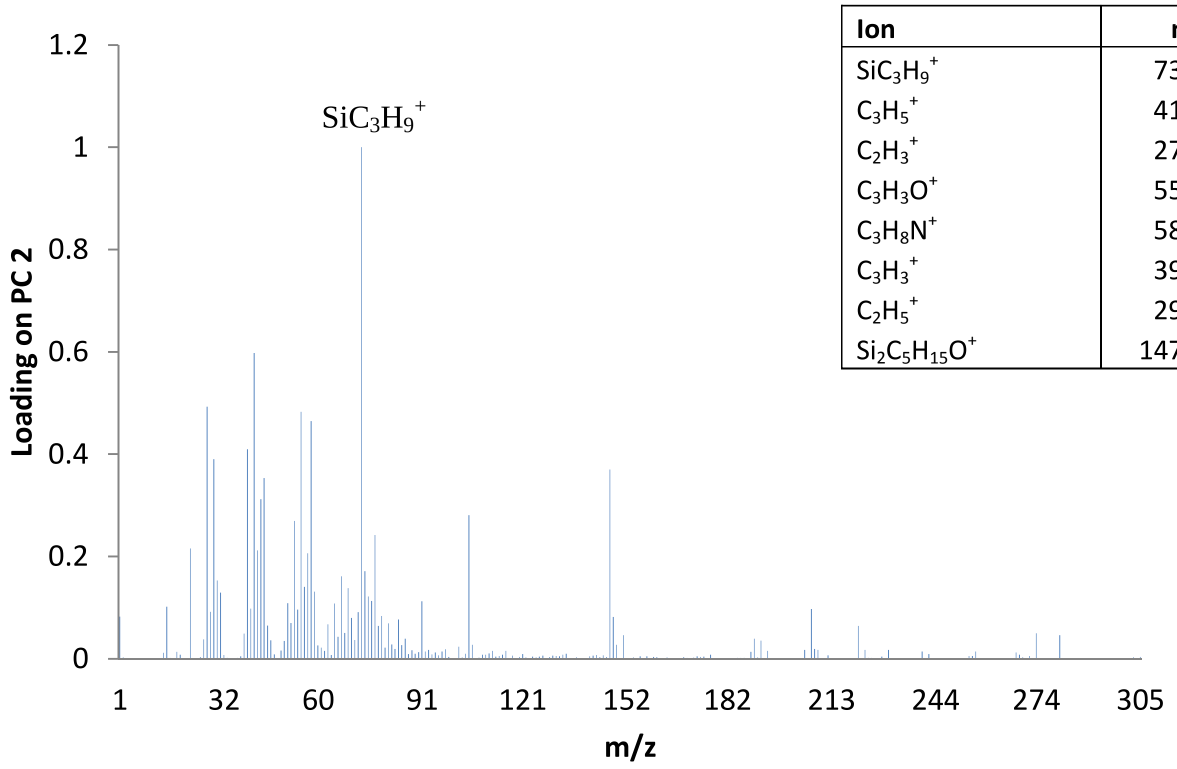

Ion	m/z
$SiC_3H_9^+$	73.06
$C_3H_5^+$	41.04
$C_2H_3^+$	27.02
$C_3H_3O^+$	55.02
$C_3H_8N^+$	58.07
$C_3H_3^+$	39.02
$C_2H_5^+$	29.04
$Si_2C_5H_{15}O^+$	147.08

SI. 2c) PC 3

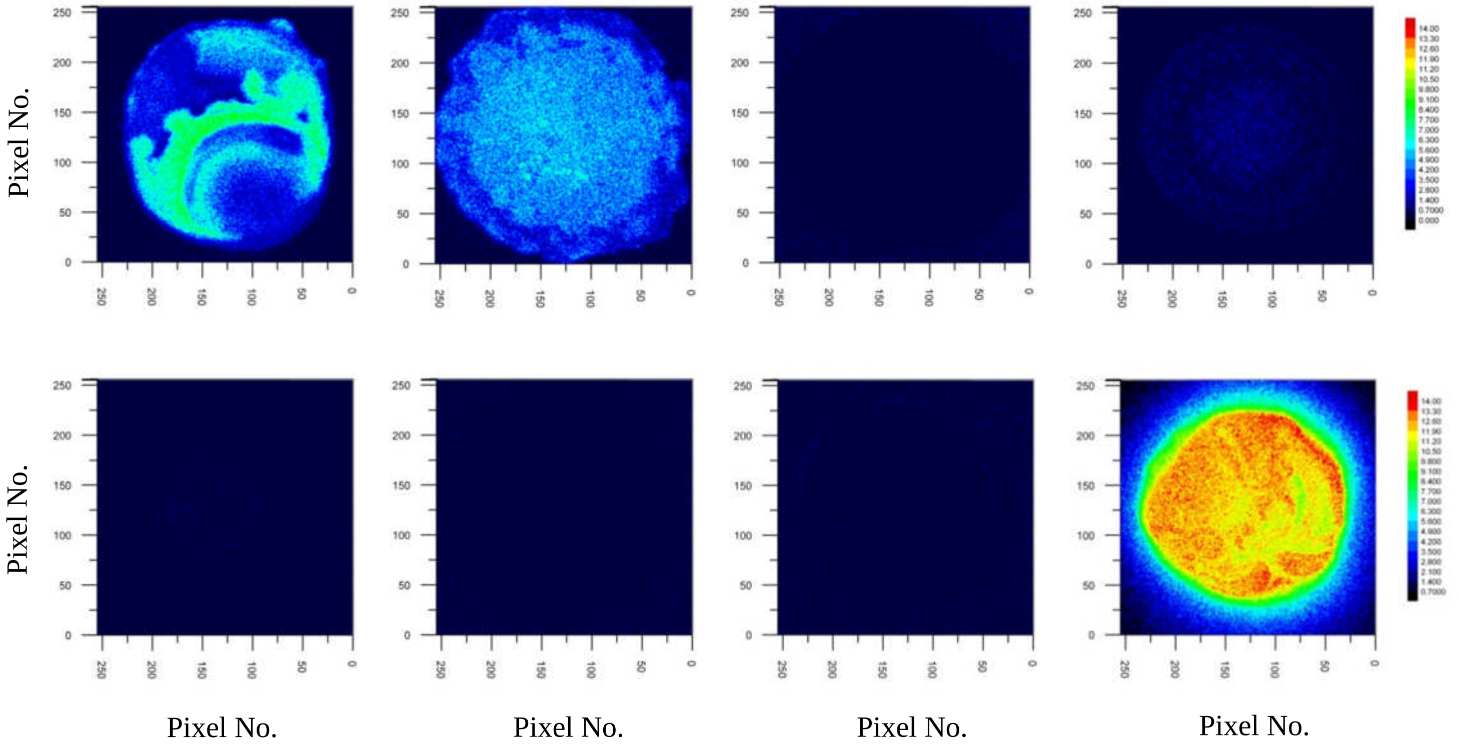

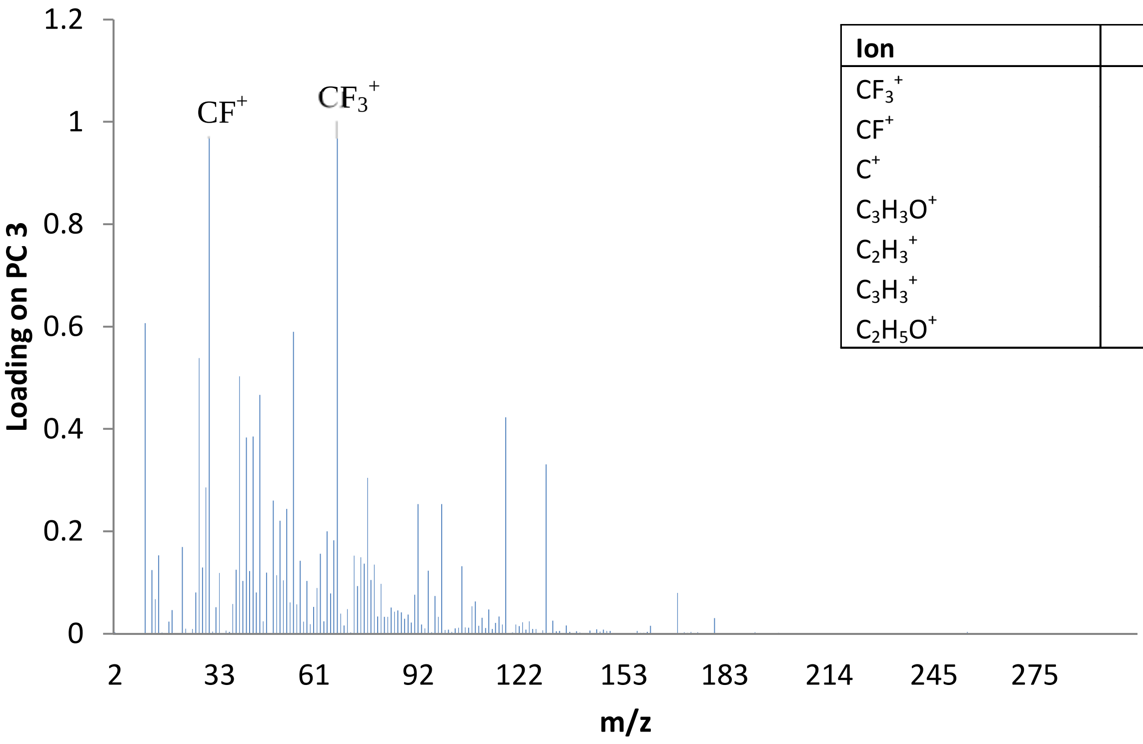

Ion	m/z
CF_3^+	69.00
CF^+	31.00
C^+	12.00
$C_3H_3O^+$	55.02
$C_2H_3^+$	27.02
$C_3H_3^+$	39.02
$C_2H_5O^+$	45.04

SI. 2d) PC 4

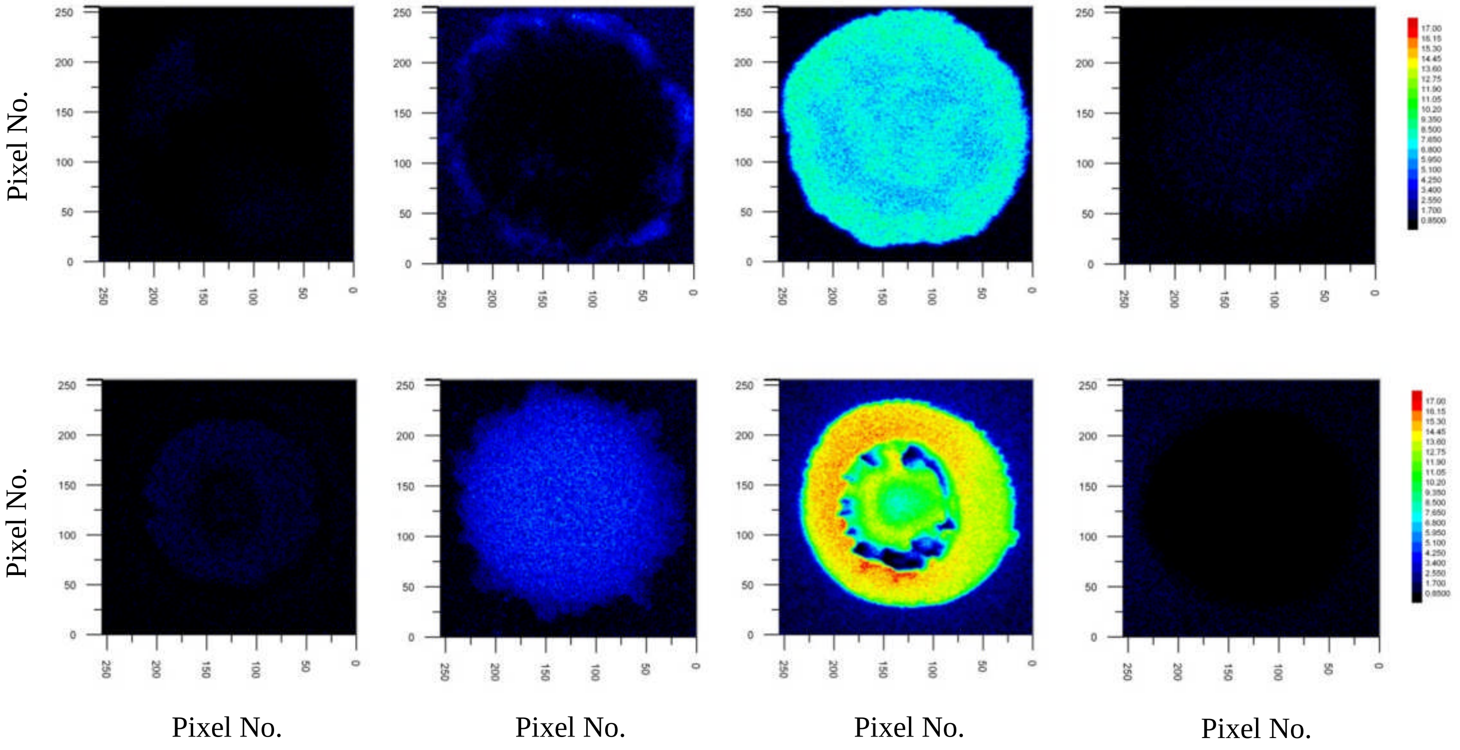

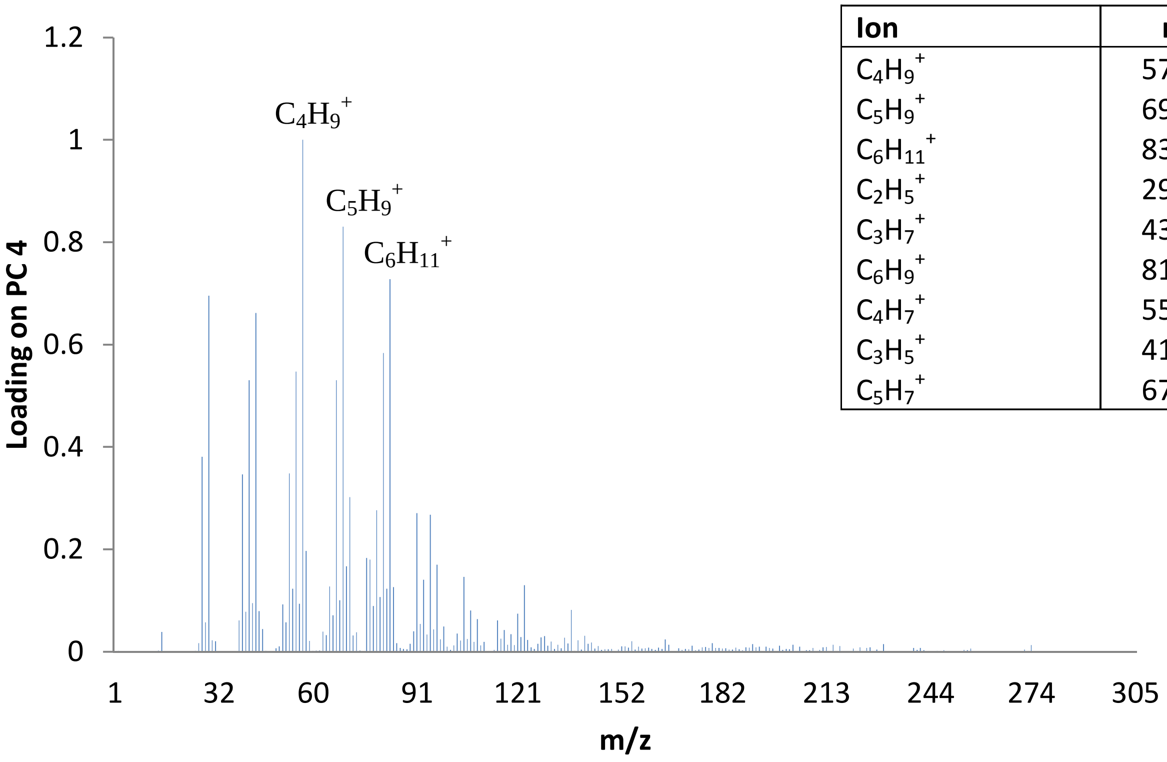

Ion	m/z
$C_4H_9^+$	57.07
$C_5H_9^+$	69.07
$C_6H_{11}^+$	83.09
$C_2H_5^+$	29.04
$C_3H_7^+$	43.06
$C_6H_9^+$	81.08
$C_4H_7^+$	55.06
$C_3H_5^+$	41.04
$C_5H_7^+$	67.06

SI. 2e) PC 5

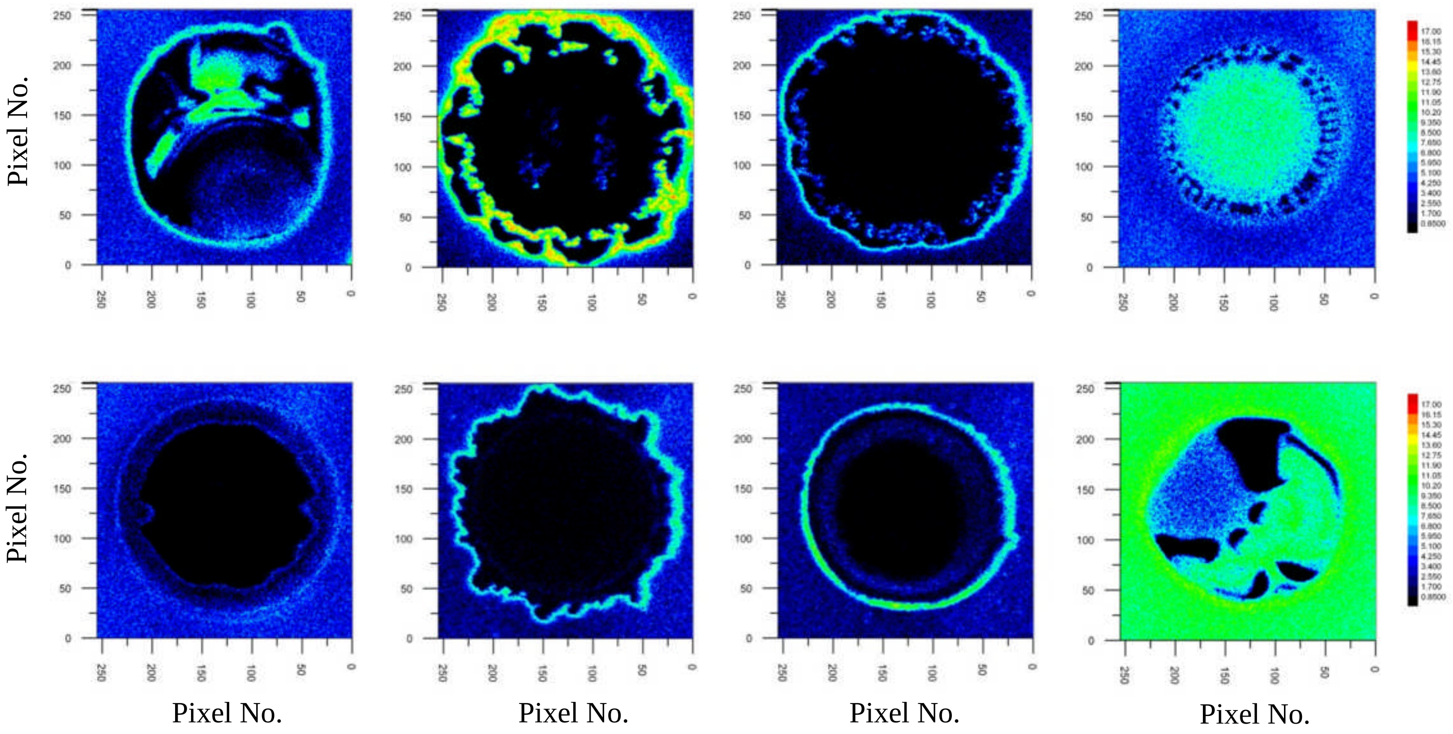

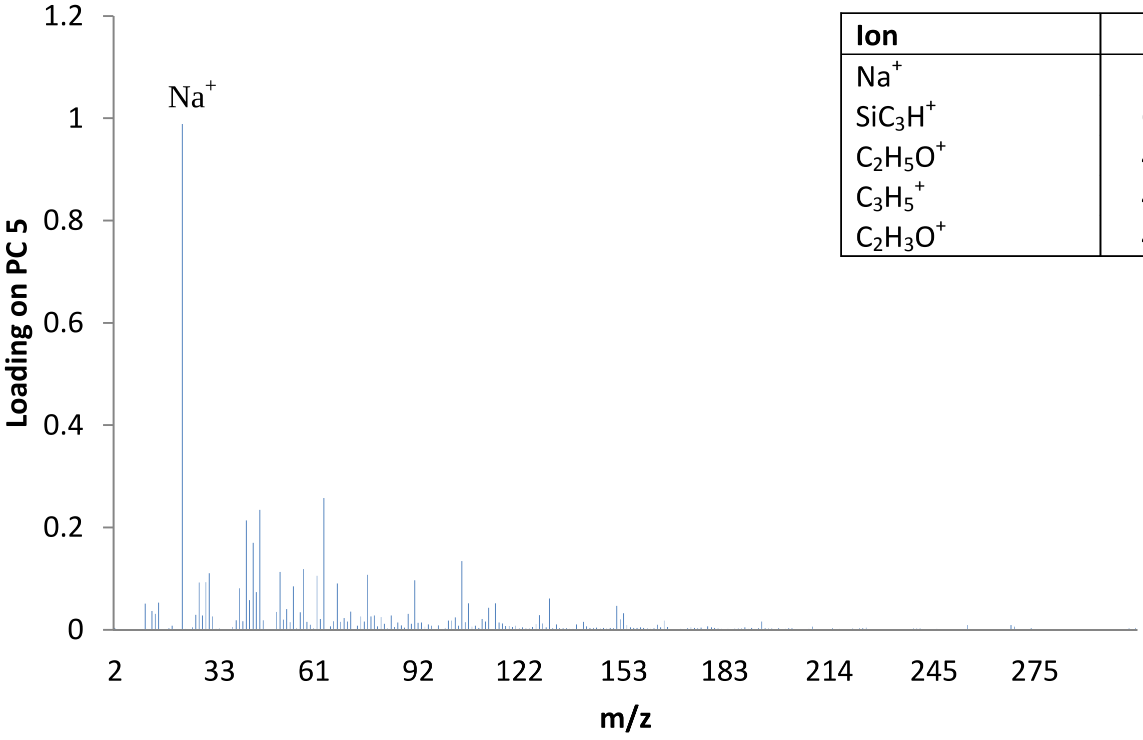

Ion	m/z
Na^+	22.99
SiC_3H^+	64.98
$C_2H_5O^+$	45.04
$C_3H_5^+$	41.04
$C_2H_3O^+$	43.02

SI. 2f) PC 6

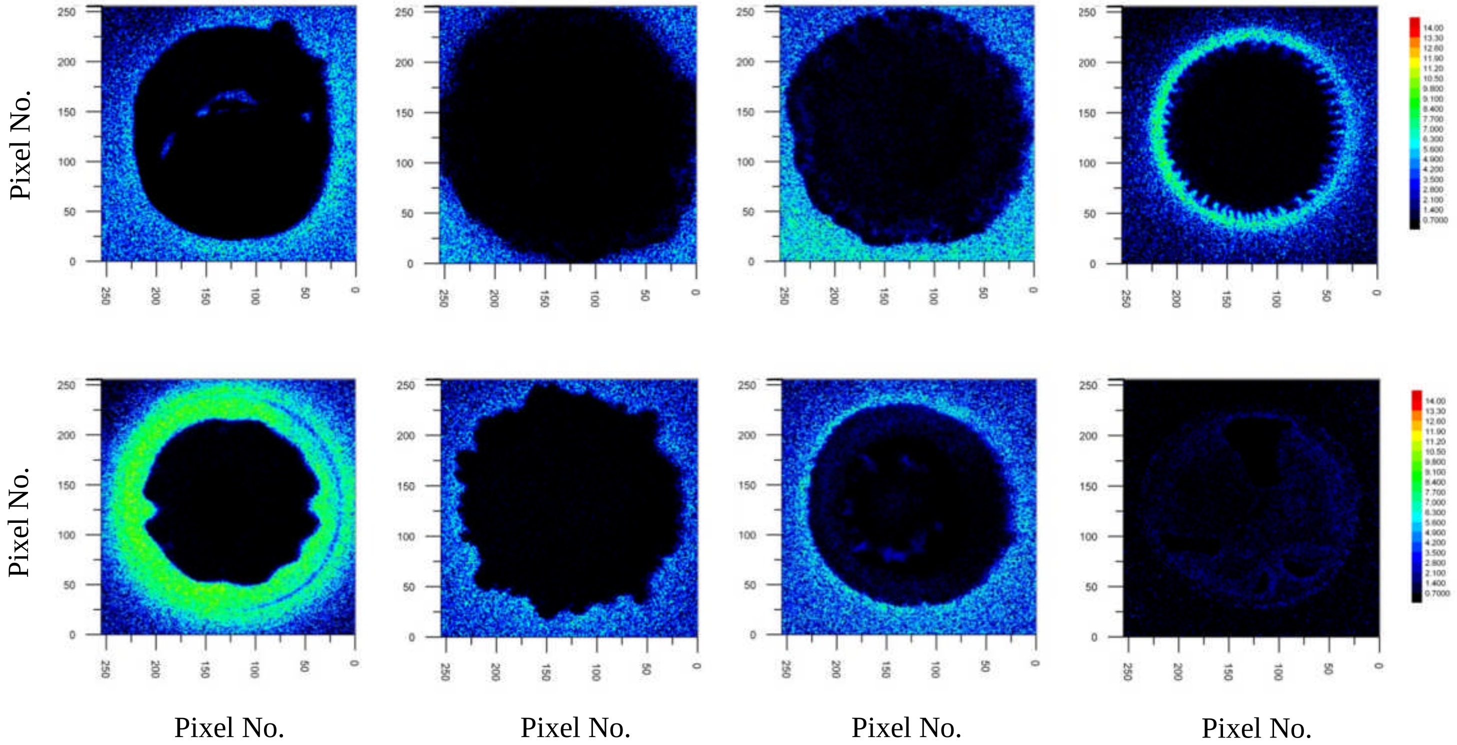

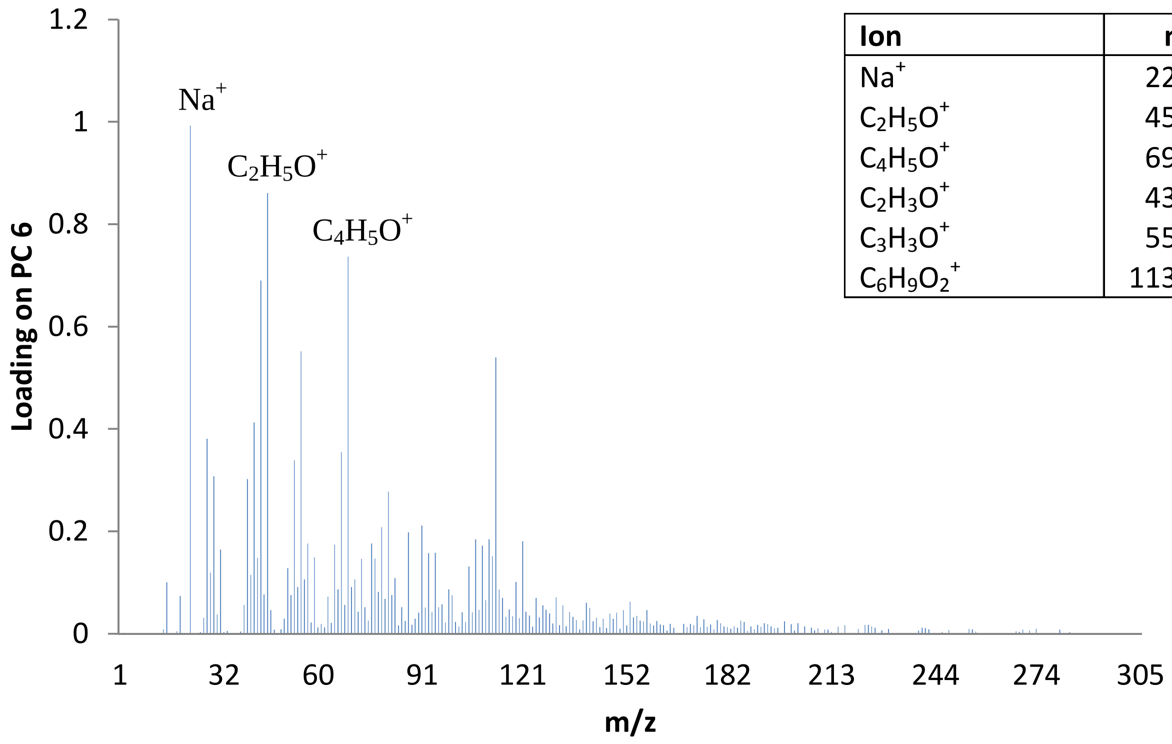

Ion	m/z
Na^+	22.99
$C_2H_5O^+$	45.04
$C_4H_5O^+$	69.04
$C_2H_3O^+$	43.02
$C_3H_3O^+$	55.02
$C_6H_9O_2^+$	113.07

SI. 2g) PC 7

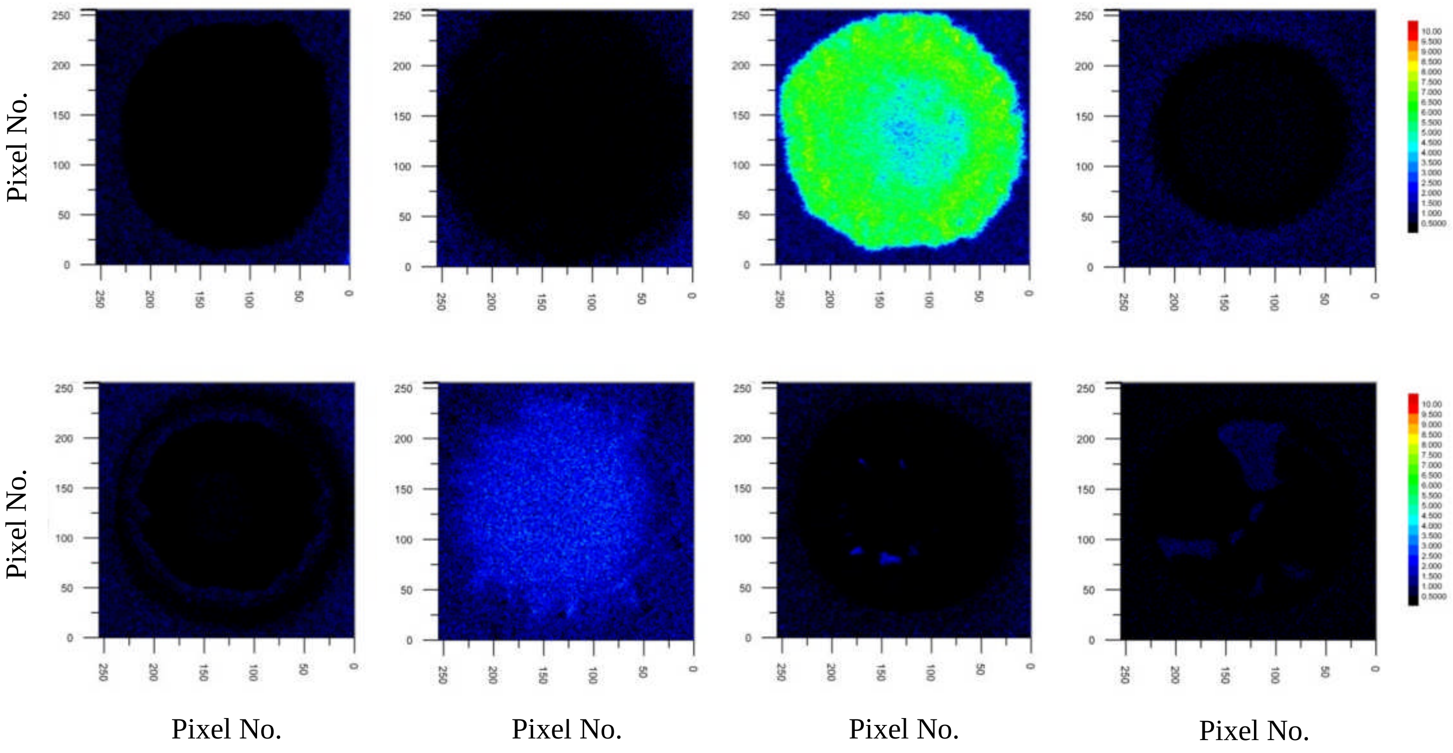

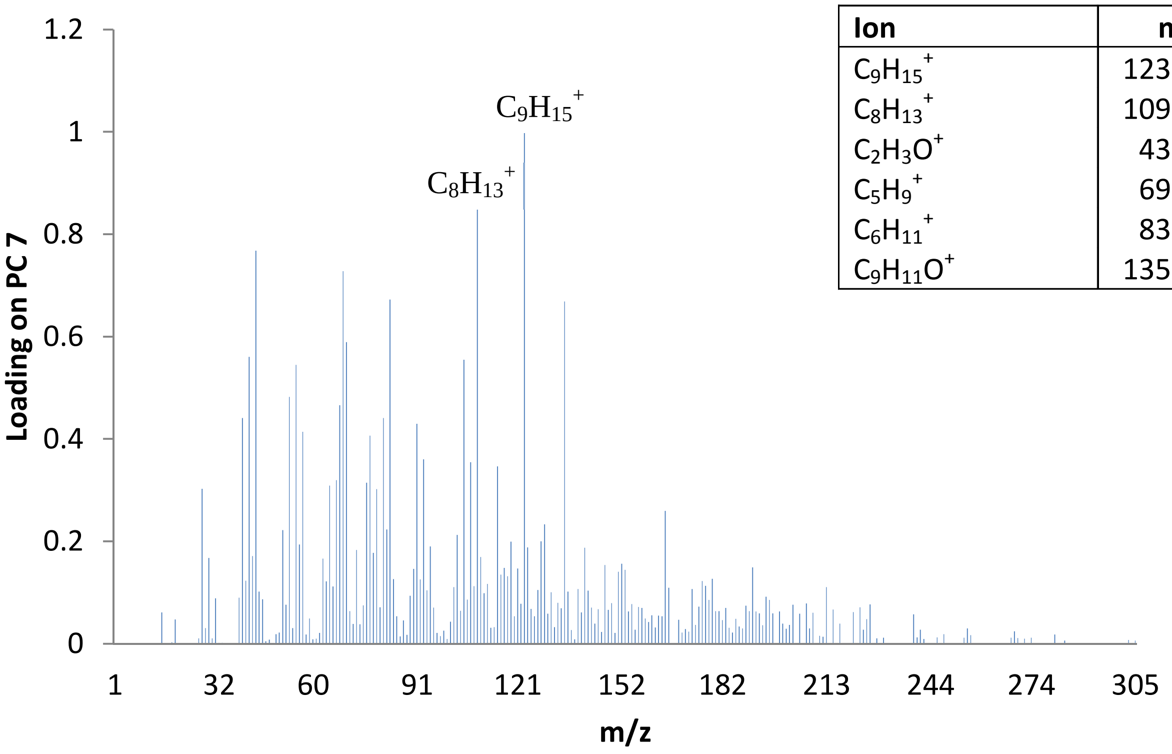

Ion	m/z
$C_9H_{15}^+$	123.13
$C_8H_{13}^+$	109.11
$C_2H_3O^+$	43.02
$C_5H_9^+$	69.07
$C_6H_{11}^+$	83.09
$C_9H_{11}O^+$	135.09

SI. 2h) PC 8

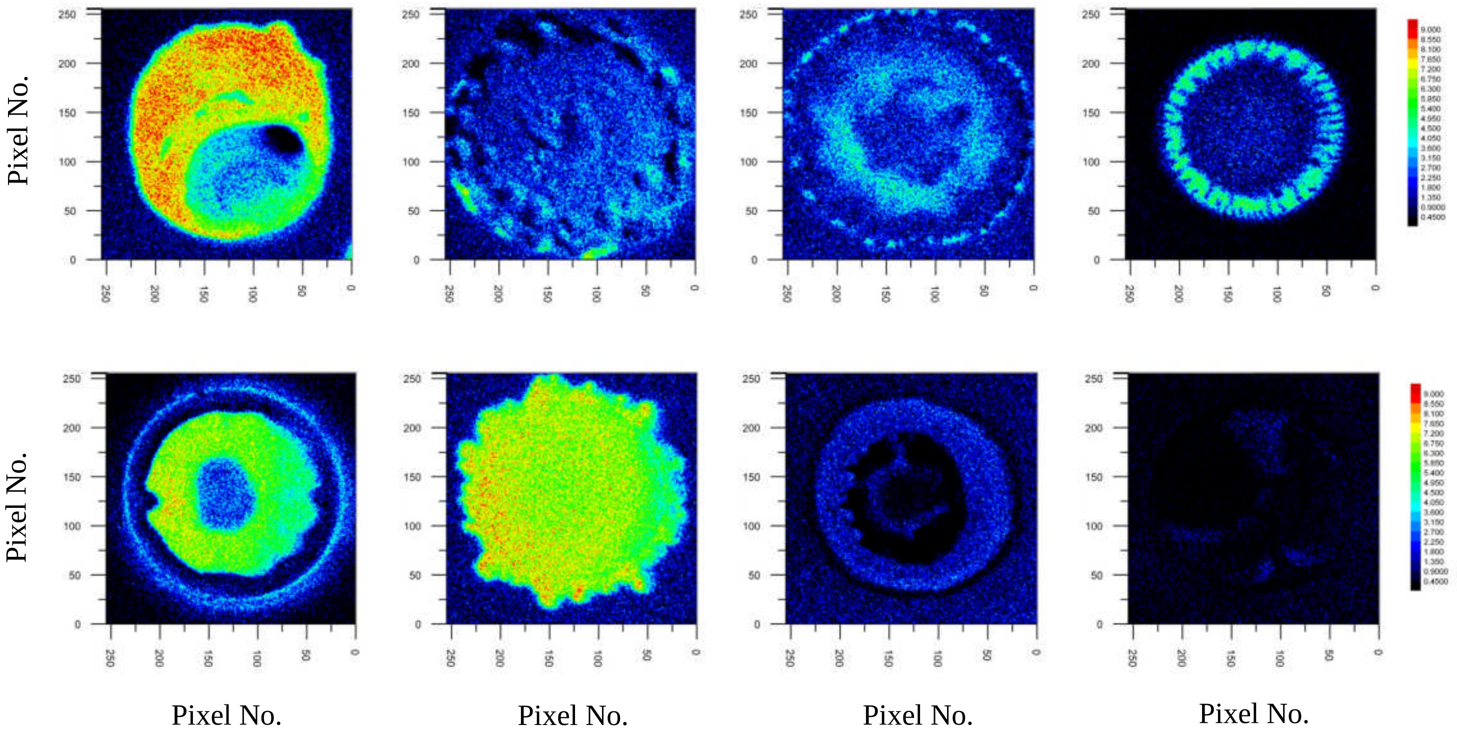

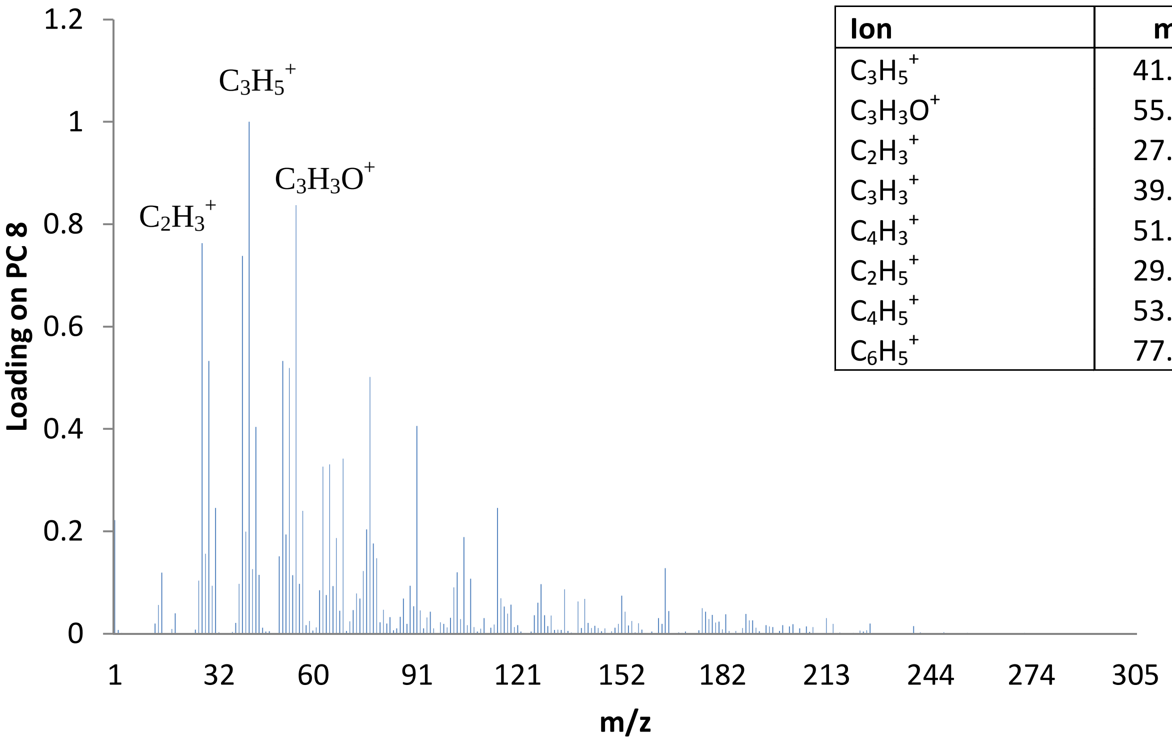

Ion	m/z
$C_3H_5^+$	41.04
$C_3H_3O^+$	55.02
$C_2H_3^+$	27.02
$C_3H_3^+$	39.02
$C_4H_3^+$	51.02
$C_2H_5^+$	29.04
$C_4H_5^+$	53.04
$C_6H_5^+$	77.04

SI. 2i) PC 9

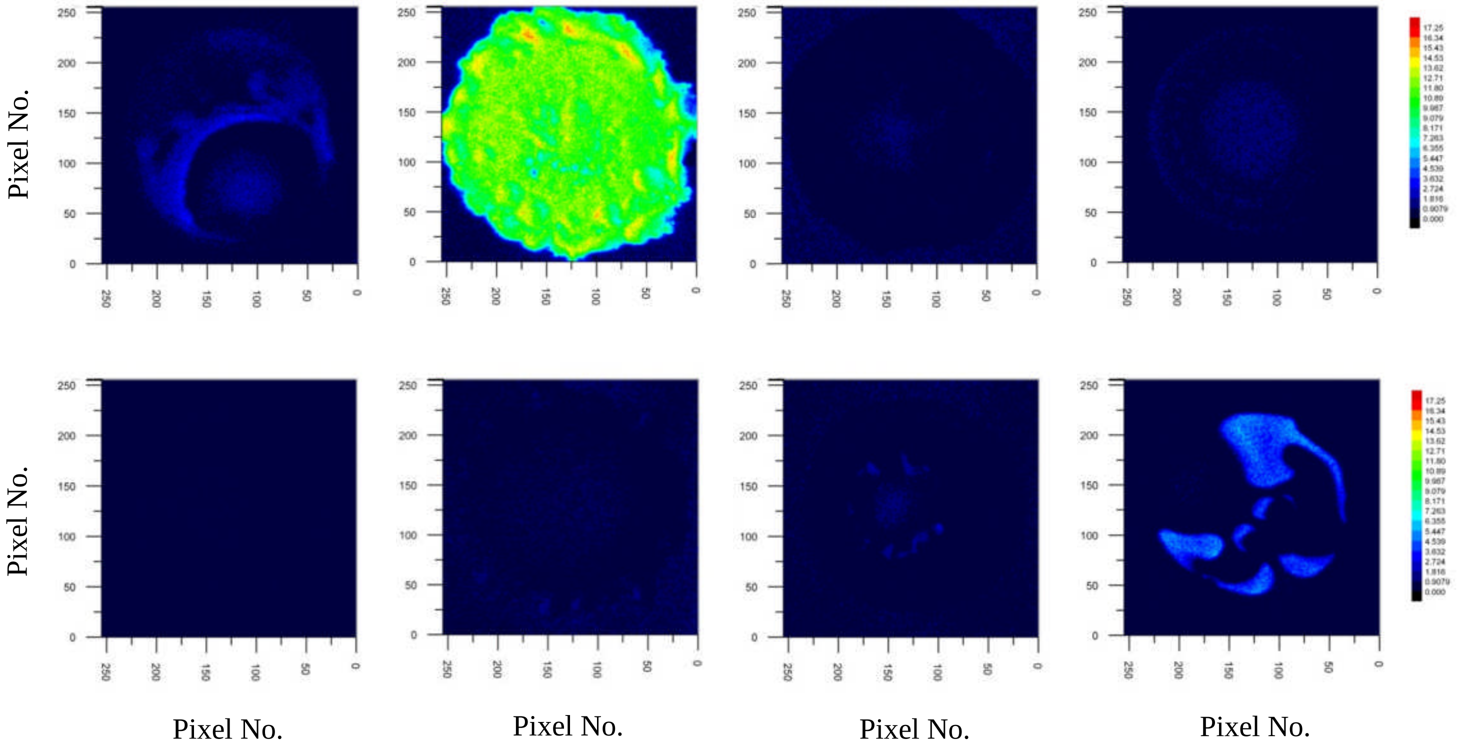

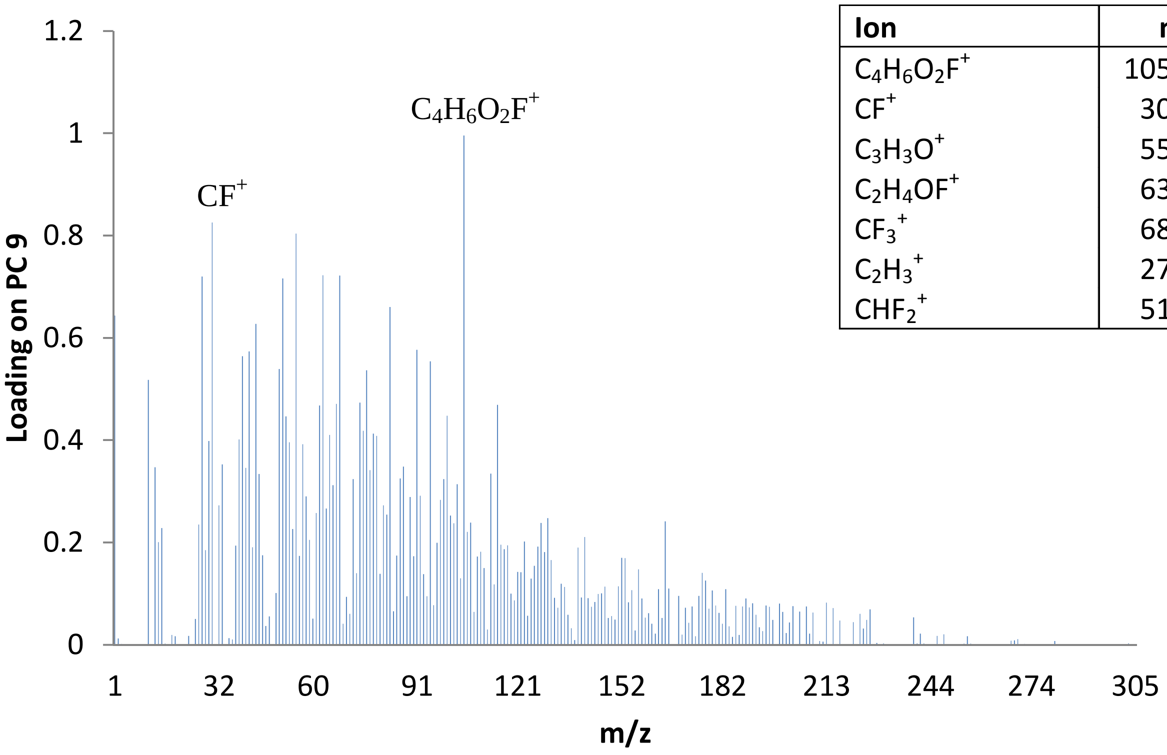

Ion	m/z
$C_4H_6O_2F^+$	105.04
CF^+	30.99
$C_3H_3O^+$	55.02
$C_2H_4OF^+$	63.02
CF_3^+	68.99
$C_2H_3^+$	27.02
CHF_2^+	51.00
