# Supplementary figures and images for: Strategies for MCR image analysis of large hyperspectral data-sets
Source: Surf Interface Anal. 2012 May 22;45(1):466–70. doi: 10.1002/sia.5040 (PMC3579489; doi:10.1002/sia.5040)

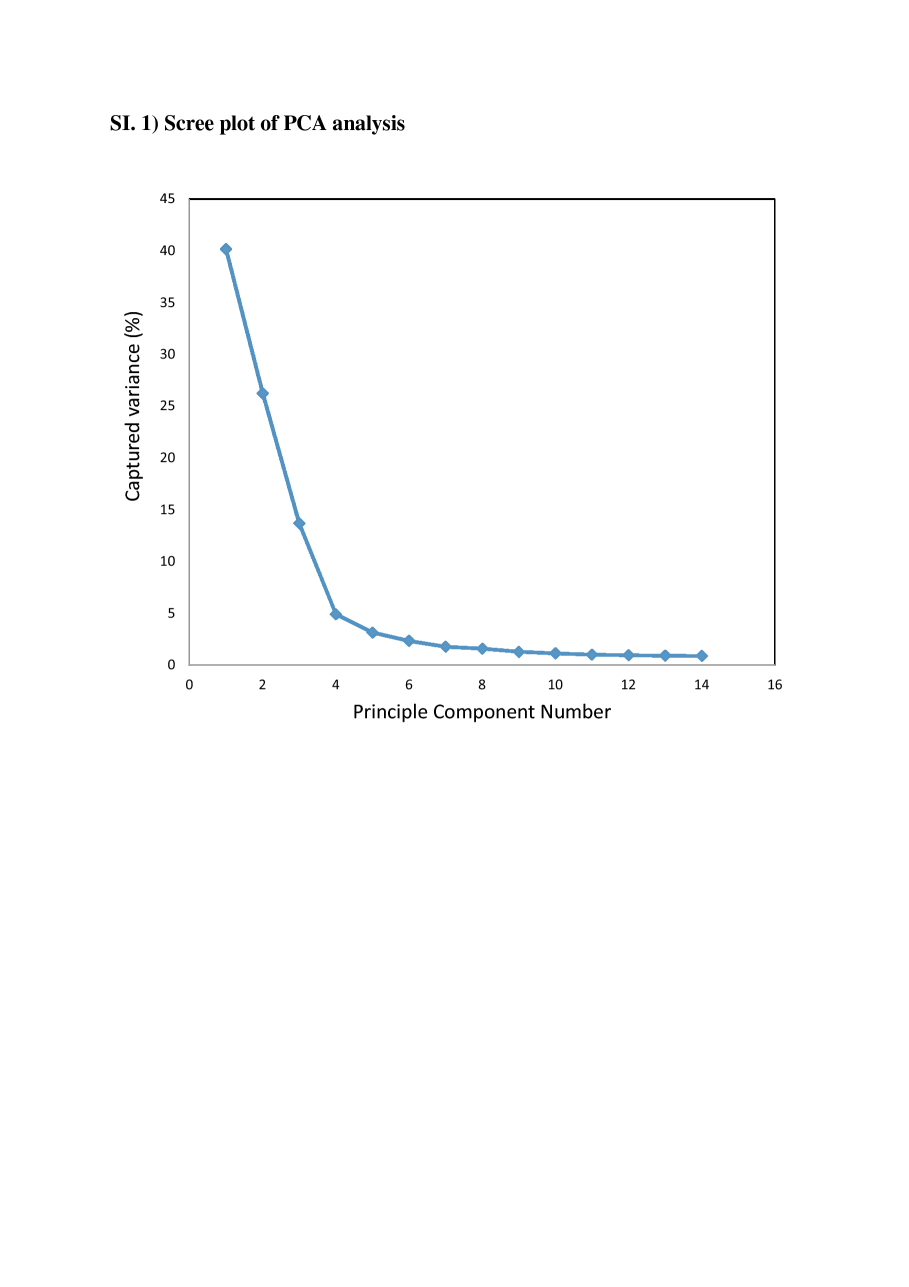

Supplement: Supplementary file 2 [file sia0045-0466-SD2.png]
